# Gene expression profile of HCT-8 cells following single or co-infections with *Cryptosporidium parvum* and bovine coronavirus

**DOI:** 10.1038/s41598-023-49488-1

**Published:** 2023-12-13

**Authors:** Alejandro Jiménez-Meléndez, Ruchika Shakya, Turhan Markussen, Lucy J. Robertson, Mette Myrmel, Shokouh Makvandi-Nejad

**Affiliations:** 1 Department of Paraclinical Sciences (PARAFAG), Faculty of Veterinary Medicine, Norwegian University of Life Sciences (NMBU), Ås, Norway; 2 Research Group Animal Health, Vaccinology, Norwegian Veterinary Institute, Ås, Norway; 3 Present Address: Nykode Therapeutics ASA, Oslo Science Park, Oslo, Norway

**Keywords:** Parasite biology, Parasite host response, Virus-host interactions

## Abstract

Among the causative agents of neonatal diarrhoea in calves, two of the most prevalent are bovine coronavirus (BCoV) and the intracellular parasite *Cryptosporidium parvum*. Although several studies indicate that co-infections are associated with greater symptom severity, the host–pathogen interplay remains unresolved. Here, our main objective was to investigate the modulation of the transcriptome of HCT-8 cells during single and co-infections with BCoV and *C. parvum*. For this, HCT-8 cells were inoculated with (1) BCoV alone, (2) *C. parvum* alone*,* (3) BCoV and *C. parvum* simultaneously. After 24 and 72 h, cells were harvested and analyzed using high-throughput RNA sequencing. Following differential expression analysis, over 6000 differentially expressed genes (DEGs) were identified in virus-infected and co-exposed cells at 72 hpi, whereas only 52 DEGs were found in *C. parvum*-infected cells at the same time point. Pathway (KEGG) and gene ontology (GO) analysis showed that DEGs in the virus-infected and co-exposed cells were mostly associated with immune pathways (such as NF-κB, TNF-α or, IL-17), apoptosis and regulation of transcription, with a more limited effect exerted by *C. parvum*. Although the modulation observed in the co-infection was apparently dominated by the virus, over 800 DEGs were uniquely expressed in co-exposed cells at 72 hpi. Our findings provide insights on possible biomarkers associated with co-infection, which could be further explored using in vivo models.

## Introduction

Neonatal diarrhoea affects calves worldwide^1,2^, adversely impacting animal welfare and leading to vast economic losses. Although calf diarrhoea can be attributed to non-infectious factors (e.g., diet and environment)^3,4^), several enteric pathogens may also be involved, such as enterotoxigenic *Escherichia coli*, *Cryptosporidium* spp., bovine rotavirus, and bovine coronavirus (BCoV)^5^. *Cryptosporidium parvum* is responsible for severe profuse diarrhoea in calves, which can lead to dehydration and death and can also have long-term health effects, such as reduced weight gain and respiratory disease later in life^6,7^. BCoV, a β-coronavirus, is another prevalent cattle pathogen^8^, causing respiratory and diarrhoeal diseases in calves and winter dysentery in adult cattle^9,10^.

Both BCoV and *C. parvum* infect enterocytes in the small intestines of calves^11,12^. Pathogens involved in the calf neonatal diarrhoea complex frequently occur as co-infections^3,13,14^. Although several studies indicate that BCoV and *C. parvum* co-infections are associated with greater symptom severity^15,16^, the host–pathogen and pathogen-pathogen interplays remain mainly unknown. For exploration of the pathogenesis in such infections and the host–pathogen interplay, in vitro models offer several advantages over costly and cumbersome in vivo models; environmental factors can be controlled, and several experimental replicates are easily achievable. In vitro transcription studies of *C*. *parvum*, investigating its pathogenicity at early stages of infection, have revealed modulation of pathways such as cell cycle^17^ and interferon type I (IFN-I) responses^17–21^. However, compared with other apicomplexan parasites, *Cryptosporidium* might exert a more limited stimulation of host immune responses due to its unique location, being intracellular but extracytoplasmic^22^. Furthermore, *C. parvum,* depends on the host-cell carbon metabolism cycle for nutrient acquisition, as previously described for other related Apicomplexa, such as *Toxoplasma gondii*^23–25^. In comparison with other apicomplexan parasites, *C. parvum* has a more restricted metabolism, lacking functional mitochondria and with various metabolic pathways lost during evolution, relying entirely on host glycolysis for nutrient acquisition^26^. In contrast, viruses, including coronaviruses, are known to exert a broad effect on host cellular responses, involving, among others, stimulation of immune mechanisms mediated by IFN-I. However, they have also evolved mechanisms to counteract the host antiviral responses^27^. To our knowledge, there have been no in vitro studies on the host transcriptome in response to BCoV infection. Despite the prevalence of co-infections with *C. parvum* and BCoV, there is only one in vitro study on the co-infection. In that study, interactions between the two pathogens were reported, with increased entry of the virus into host HCT-8 cells when the pathogens were co-inoculated^28^. However, the host immune response to the co-infection was not investigated.

Here, our aim was to compare the differential gene expression profiles of HCT-8 cells following single and co-infections with *C. parvum* and BCoV by means of high-throughput RNA sequencing.

## Materials and methods

### Cell culture

A human ileocecal colorectal adenocarcinoma cell line (HCT-8, ATCC CCL-244) was kindly provided by Professor Elisabeth A. Innes and Alison Burrells (Moredun Research Institute, Scotland). The cells were grown in T75 Nunc flasks (Thermo Fisher Scientific, Grand Island, NY, USA) in RPMI 1640 culture medium (Thermo Fisher Scientific) supplemented with 10% foetal bovine serum (FBS) (Thermo Fisher Scientific), 2% L-glutamine, and 1% Penicillin Streptomycin (PenStrep) (Life Technologies, Paisley, Scotland). Culture of cells and incubation/culture of pathogens were performed in a humidified incubator at 37 °C with 5% CO_2_. After inoculation, a maintenance medium, consisting of RPMI 1640 with 2% FBS, 2% L-glutamine and 1% PenStrep, was employed. The HCT-8 cells used in the experiments were from passage 16–20 and certified as free from *Mycoplasma* spp*.* by PCR at a commercial facility (Eurofins Genomics).

### Bovine coronavirus (BCoV)

The BCoV strain used in this study was originally isolated from a calf faecal sample and adapted to the human rectal tumour cell line, HRT-18G^9^. This isolate was further adapted to HCT-8 cells during several passages^28^, and the supernatant titrated in a 96-well plate by ten-fold serial dilutions, using the Spearman-Karber method^29^ (TCID_50_/mL = 1.0E + 07), aliquoted and stored at -80 °C.

### Excystation of *Cryptosporidium parvum*

*C. parvum* oocysts (Iowa-II strain) were obtained from Bunch Grass Farm (Deary, ID, USA) and used within one month of receipt. Prior to excystation, the viability was assessed by inclusion/exclusion of 4’,6-diamino-2-phenylindole/propidium iodide (DAPI/PI), as previously described by Campbell et al.^30^.

The oocysts were pre-treated with 6% sodium hypochlorite and excysted as previously described^28,31^. Briefly, oocysts were washed, treated with 0.05% trypsin–EDTA (Life Technologies) with 2% HCl (Merck Life Sciences, Darmstadt, Germany), then placed in a water bath at 37 °C for 30 min. After washing, the pellet was resuspended in PBS with 2.2% sodium bicarbonate (Merck Life Sciences) and 1% sodium taurodeoxycholate (Sigma-Aldrich, St. Louis, MO, USA). After incubation in a 37 °C water bath for 1 h, the excysted sporozoites were resuspended in RPMI 1640 medium without FBS and passed through a 3 μm filter (Merck Millipore) to remove oocyst shells and non-excysted oocysts. The excystation rate was determined by relating the number of oocyst shells plus partially excysted oocysts to the total number of oocysts and calculated to be 85–90%. The motility of the sporozoites was observed by microscopy, and intactness analysed by trypan blue exclusion.

### Cell inoculation

Inoculation of HCT-8 cells was performed in T25 flasks (Sarstedt, Nümbrecht, Germany) seeded with 3E + 05 cells. The cells were grown to 60% confluency (estimated by microscopy and achieved after 3 days of culture, and cell number estimated to be 1.2E + 06), washed with PBS, and inoculated with: i) RPMI without FBS (uninfected control), ii) BCoV, iii) *C. parvum* sporozoites, and iv) *C. parvum* sporozoites together with BCoV. The inocula were kept on the cells for 2 h in a 37 °C humidified incubator before washing and adding maintenance medium. The selected multiplicity of infection (MOI) was four for *C. parvum* sporozoites (1.2E + 06 oocysts/flask, assuming one viable oocyst contains 4 sporozoites), and one for BCoV (1.2E + 06 TCID_50_).

Each of the four experimental groups consisted of 12 replicates, of which six were harvested at 24 h post inoculation (hpi) and six at 72 hpi, giving a total of 48 samples. These time points were selected based on previous studies and should be representative of the first round of asexual replication (24 hpi) and differentiation into gametes (72 hpi) according to Tandel et al. (2019) for *Cryptosporidium parvum*. For BCoV, by 24 hpi the virus would have completed the first round of replication, and 72 hpi was selected to avoid CPE for the strain employed (personal observations)..Harvesting for RNA-Seq was by direct lysis of the cells by addition of 1.2 mL of RLT buffer plus DTT (2 M) after washing with cold PBS. Lysates were kept at -80 °C until RNA extraction.

In parallel, HCT-8 cells grown on 12 mm glass coverslips in 24-well plates were seeded (43,000 cells/well) and inoculated at 60% confluency in the same way to visualize infected cells by immunostaining.

### Immunostaining

Following removal of media, the cells were washed twice with cold Dulbecco’s PBS with 0.05% sodium azide and 2% bovine serum albumin (BSA) (Merck Life Sciences). The cells were fixed with IC Fixation buffer (eBioscience, 131 San Diego, CA, USA) at room temperature for 20 min, washed with permeabilization buffer (eBioscience), and blocked using PBS with 2% BSA and 0.1% Tween 20. To stain for BCoV or *C. parvum*, monoclonal mouse anti-BCoV antibodies (1:80) labelled with fluorescein isothiocyanate (FITC; Bio-X Diagnostics, Rochefort, Belgium) or 1 × Cy3-labelled polyclonal Sporoglo™ antibody (Waterborne Inc., New Orleans, LA, USA), (1:20) in permeabilization buffer (eBioscience) were used, with co-exposed cells receiving both primary antibodies. After 1 h incubation in the dark, the cells were washed, counterstained with Hoechst 33,342 (1:10,000) (Invitrogen, Waltham, MA, USA), and the coverslips transferred onto slides with Fluoroshield (Sigma-Aldrich, St. Louis, MO, USA). The slides were examined using a Leica Inverted Confocal SP5 equipped with a White Light Laser, a Leica HyD Detector (Leica microsystems GmbH, Mannheim, Germany). A minimum of 10 fields were examined per slide, and images were captured at 40 × magnification under oil immersion using the Leica Application Suite software.

### Isolation of total RNA

Total RNA was isolated from the cell lysates using QIAGEN Rneasy Mini Plus Kit (Qiagen, Hilden, Germany), following the manufacturer’s instructions, including a gDNA elimination step. RNA concentration was determined using NanoDrop ND-1000 spectrophotometer (ThermoFisher Scientific, Waltham, Massachusetts, USA). The RNA integrity number (RIN) and size distribution were assessed using Bioanalyzer 2100 (Agilent Technologies, Santa Clara, CA, USA). Samples with concentrations of RNA between 0.5 and 5 μg/mL, RINs higher than 9, and 260/280 absorbance ratios higher than 2.0 were included in the study. Samples were normalized to 400 ng/µL before library synthesis.

### Transcriptome sequencing, assembly, annotation, and statistical analysis

Transcriptome libraries were prepared by the Norwegian Sequencing Center (https://www.Sequencing.uio.no/) using a TruSeq® Stranded mRNA Library Prep kit, following the manufacturer’s protocol. Stranded sequencing was performed on Novaseq 6000 with paired end sequencing at 50 bp, using a Novaseq S1 full flow cell. Following read quality assessment by FastQC (www.bioinformatics.babraham.ac.uk/projects/fastqc/), Trimmomatic was used for quality assessment and trimming low quality bases in order to retain high quality^32^. Reads of low quality (Phred score < 30), low complexity, containing adapter sequences, or with sequences matching ribosomal or mitochondrial RNA, were discarded. Reads were mapped to the CRGh38/hg38 assembly using TopHat (version 2.0.13)^33^ and reads with more than a single hit in the genome were discarded. Cufflinks^34,35^ was used to generate transcriptome assemblies for each sequenced sample and merged by Cuffmerge to construct a single gene transfer file. Expression data were normalized via the median of geometric means of fragment counts across all samples, where relative expressions are expressed as fragments per kilobase of exon per million mapped reads (FPKM) values. Cuffdiff was then used to estimate the expression abundances of the assembled genes and transcripts and to test for differential levels of expression between groups (exposed vs non-exposed and single-exposed vs co-exposed). Transcripts with > 1.5-fold difference in expression and corrected *p*-values (q-values, false discovery rate (FDR) adjusted) of < 0.05 were assigned as differentially expressed (DE). The heatmaps illustrating the differentially expressed genes (DEGs) were plotted in RStudio (Version 1.4.1103). For further analysis, only genes that showed no overlapping in infected and uninfected cells replicates were considered.

To assess reproducibility and experimental variation among biological replicates, RNA-Seq data was subjected to principal component analysis (PCA).

### Functional enrichment and network analysis: gene ontology and pathway enrichment

Functional enrichment was performed by assessing gene ontology (GO) terms and pathway analysis, Kyoto Encyclopaedia of Genes (KEGG)^36^, and was carried out on our list of DEGs by the online tool String version 11.5 (https://string-db.org/)^37^ and plotted in RStudio. The analysis was performed against human reference genome with q-value < 0.005 (FDR Benjamini and Hochberg method).

Due to the large number (> 6000) of DEGs in some of the comparisons and limitations of the software employed, further enrichment analyses (GO, KEGG) were performed with the 2000 genes that appeared to be most significantly regulated, either up or down, according to their fold change (FC) and p-adjusted value.

## Results and discussion

### Generation of RNA-Seq data and mapping against Homo sapiens genome

Using high throughput RNA-Seq, we generated approximately 40 million reads per sample. After alignment, an average of 86% of the high-quality reads were mapped against the reference *Homo sapiens* genome, providing a global indicator of sequencing accuracy (Table 1).

**Table 1:** RNA-sequencing and genome mapping statistics (million reads) from HCT-8 cells that were uninfected, or infected with bovine coronavirus (BCoV), *Cryptosporidium parvum* or both agents.

Post infection timepoint (hpi)	Uninfected (Controls)		BCoV		C. parvum		Co-exposed (BCoV + C. parvum)	
	24	72	24	72	24	72	24	72
Raw reads	39.883	40.364	41.346	45.148	47.200	47.672	46.339	39.948
Trimmed reads	38.905	39.453	40.034	43.703	46.070	46.652	45.294	39.046
Mapped reads (Homo sapiens) (%)	97	97	79	72	97	97	78	73

**Figure 1:**
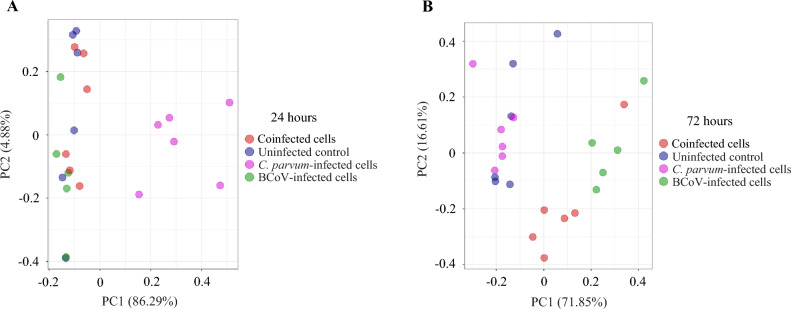
(**A**) PCA plot: Principal Component Analysis of *Homo sapiens* transcriptome at 24 hpi (**A**) and 72 hpi (**B**) Analysis based on fragments per kilobase of exon per million mapped reads (FPKM).

The PCA plot showed that, except for a few anomalies, samples belonging to different biological groups clustered together. The virus-infected and co-exposed cells clustered similarly at both 24 and 72 hpi. Uninfected cells clustered with virus-infected and co-exposed cells at 24 hpi, and clearly differed from *C. parvum*-infected cells. However, at 72 hpi, uninfected cells clustered more closely with *C. parvum-*infected cells (Fig. 1).

In addition, volcano plots illustrated the higher number of DEGs identified in virus and co-exposed cells at 72 hpi (Supplementary Fig. 1). It is important to note that, for both the single and co-infections, immunostaining (Supplementary Fig. 2) showed that the proportion of cells infected with *C. parvum* was lower than those infected with BCoV. This might have biased the results, as the gene expression profile from fewer *C. parvum-*infected cells might have been diluted by the presence of a significant majority of uninfected or virus-infected cells. A previous study using the same set up as described here found average proportions of virus-infected, *Cryptosporidium*-infected, and dual-infected HCT-8 cells by flow cytometry to be 16%, 7%, and 0.04%, respectively^28^. In this scenario, and considering the low percentage of truly coinfected cells, it is also possible that the host responses detected are due to the additive effect of cells harbouring one pathogen rather than single cells infected with both pathogens.

### Host cell transcriptome modulation by bovine coronavirus and C. parvum

#### Single infection with bovine coronavirus and co-infection with *C. parvum* produced stronger modulation of host cell transcriptome than the single infection with *C. parvum*

**Figure 2:**
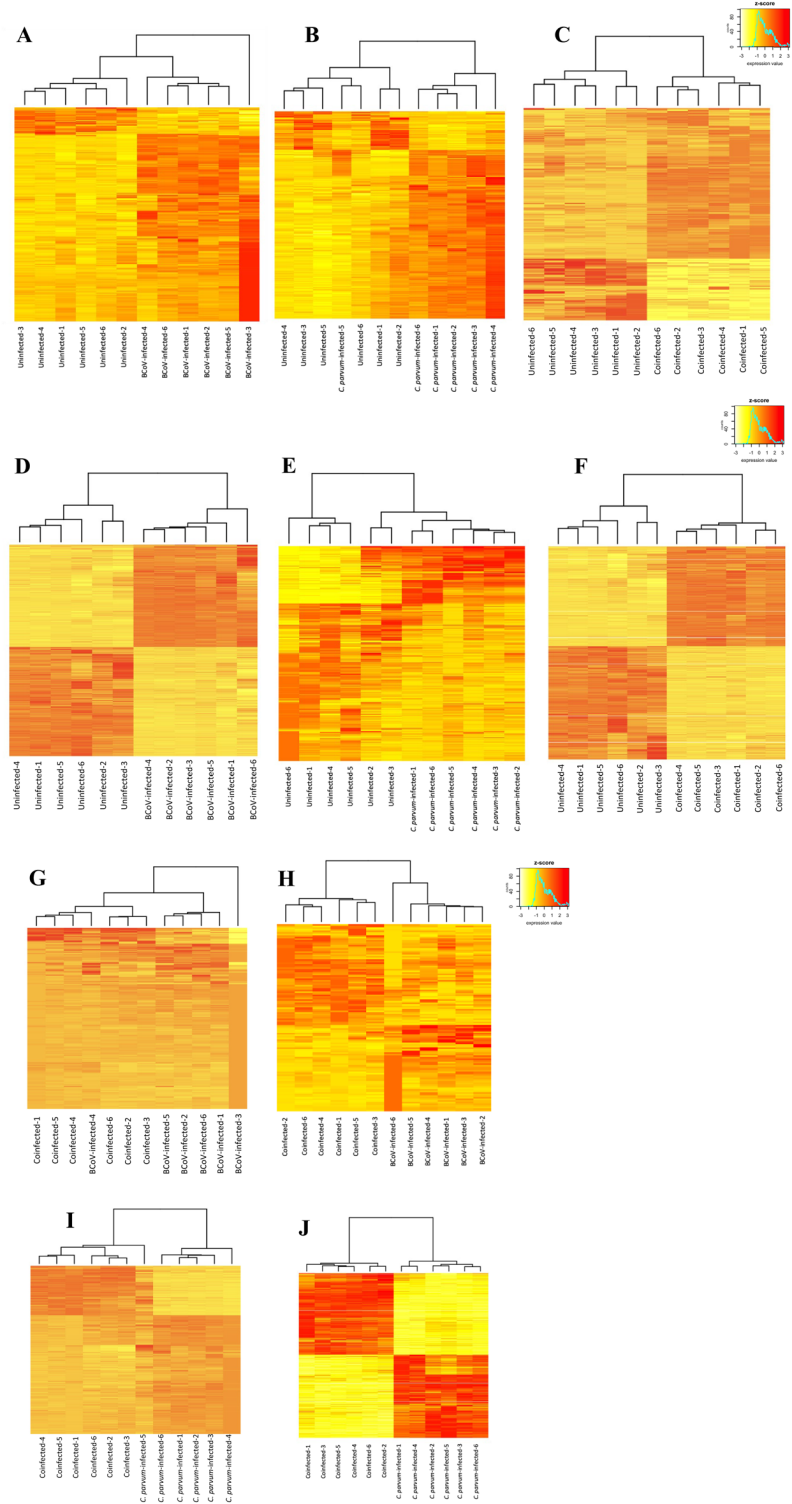
Heatmap of differentially expressed genes of *Homo sapiens* (i.e., uninfected HCT-8 cells; Control) or cells infected with BCoV and/or *C. parvum* at different time points: BCoV-infected cells at (1) 24 and (2) 72 hpi, *C. parvum-*infected cells at (3) 24 and (4) 72 hpi, Co-exposed cells at (5) 24 and (6) 72 hpi, BCoV-infected and co-exposed cells at (7) 24 hpi and (8) 72 hpi, *C. parvum* and co-exposed cells at (9) 24 and (10) 72hpi.

**Figure 3:**
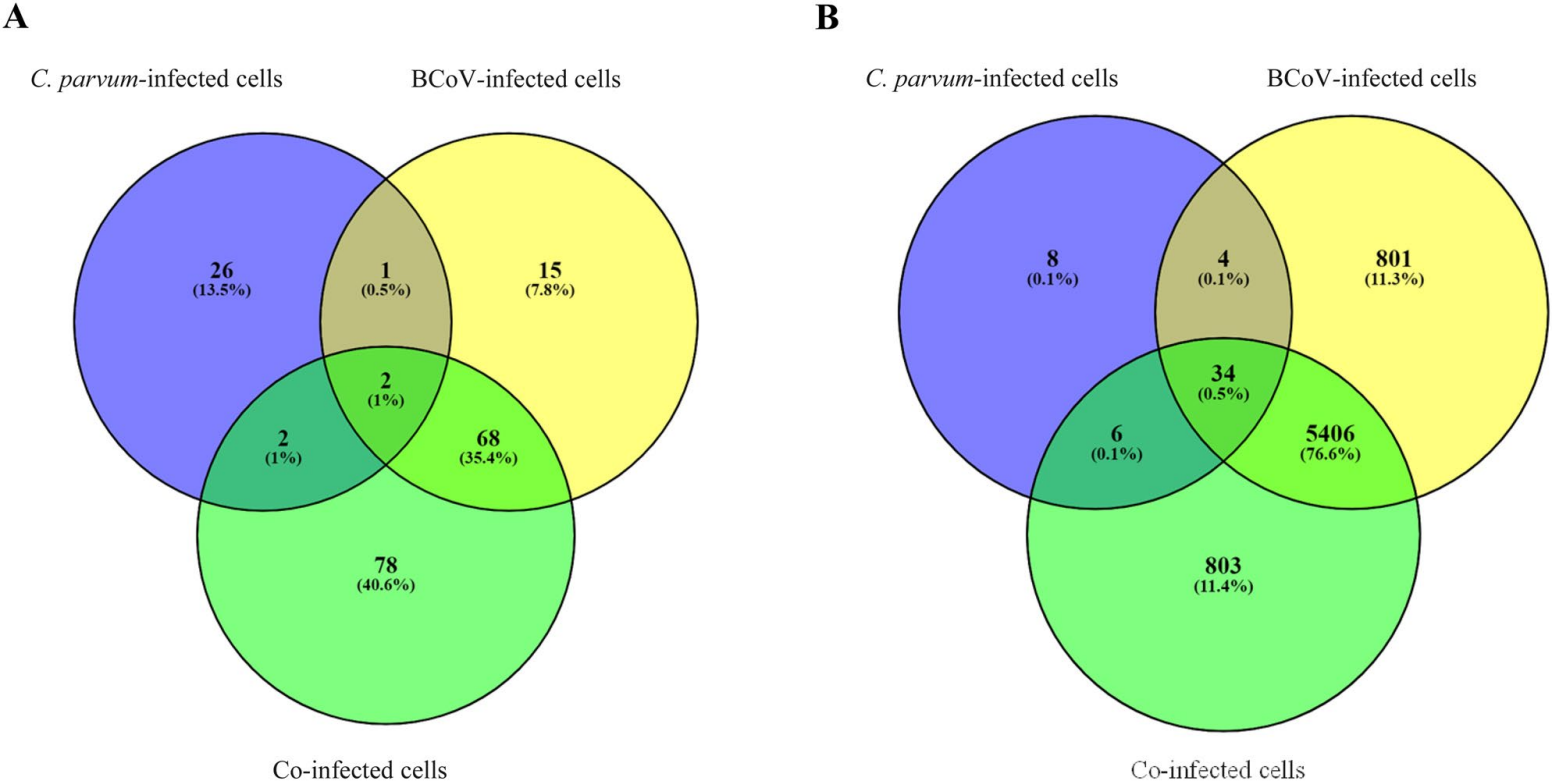
Venn diagrams with *Homo sapiens* differentially expressed genes in the different comparisons assessed in the present work at (**A**) 24 and (**B**) 72 hpi.

The host gene expression profiles for the different biological groups are reflected in the hierarchical analysis (Fig. 2). *C. parvum*-infected cells at 24 hpi showed the lowest number of DEGs (Table 2, Supplementary table 1), whereas the number of DEGs and the general gene expression profile for the BCoV-infected cells were similar to those of the co-exposed cells at 24 hpi (Table 2; Supplementary table 1; Figs. 2, 3). However, at 72 hpi, a more pronounced effect on the host responses was observed when the cells were infected with BCoV or co-exposed, with over 6000 DEGs (Supplementary table 1), visualized by the expression patterns in the cluster heatmaps (Fig. 2). In contrast, only 52 DEGs were identified in *C. parvum*-infected cells (Fig. 3). The highest FC in BCoV-infected cells at 72 hpi was for the gene coding for interleukin-8 (*IL-8*) (FC = 323.8), which was also the gene with the highest fold change in co-exposed cells (FC = 268.5). *IL-8* (also termed *CXCL-8*) is a potent chemoattractant for neutrophils^38,39^ and levels increase in infections with other coronaviruses, such as SARS-CoV-2^40^. In *C. parvum*-infected cells, the highest FC in expression was displayed by the gene encoding the Early Growth Response 1 (*EGR1*) (FC = 6.22) at 72 hpi. Increased *EGR1* expression in epithelial cells has been associated with stress-induced specific response to pathogens^41^ and has been reported to induce apoptosis in *C. parvum-*infected cells^42^.

**Table 2:** Summary of differential expressed genes (DEGs) after single or co-infections of HCT-8 cells.

Post infection time (hpi)	BCoV		*C. parvum*		Co-exposed (BCoV + *C. parvum)*		BCoV vs Co-exposed		*C. parvum* vs co-exposed	
	24	72	24	72	24	72	24	72	24	72
Differentially expressed genes (DEGs)	86	6246	31	52	150	6252	14	26	197	6252
Upregulated genes	80	3197	26	26	141	3085	5	16	165	3234
Downregulated genes	6	3049	5	26	9	3167	9	10	32	3058

When co-exposed cells were compared to single infections, there were more similarities in the gene expression profiles between BCoV-infected and co-exposed cells than between the *C. parvum*-infected and co-exposed cells, as indicated by the higher number of DEGs on the later comparison (n = 197 and 6292 at 24 and 72 hpi respectively) (Supplementary table 2. *ANKRD1* (FC = 42) was identified to be expressed with the highest FC in co-exposed cells at 24 hpi. At 72 hpi, the gene displaying the highest FC, and overexpressed in co-exposed cells, was the one encoding *IL-8* (FC = 268.5). Moreover, when DEGs from co-exposed cells were compared to those from BCoV-infected, 14 and 26 DEGs were identified in BCoV-infected cells at 24 and 72 hpi, respectively. The highest FC in transcript levels at 24 hpi in BCoV-infected cells was for *RP11-211C9.1* (FC = 1.92), and, at 72 hpi, *CTC-534B23.1* (FC = 2.3). These findings strongly suggest that BCoV infection was the main contributor to the transcriptome differences observed in the co-infection when compared to the uninfected cells. Despite the similarities shown between only virus-infected and co-exposed cells, 78 genes were identified that were only expressed in co-exposed cells at 24 hpi, and this number increased to 803 at 72 hpi (Fig. 3), as further described in section "Genes exclusively expressed in co-exposed cells as potential biomarkers for BCoV and C. parvum co-infection".

#### Gene ontology (GO) analyses show extensive modulation of host cell biological processes after infection

**Figure 4:**
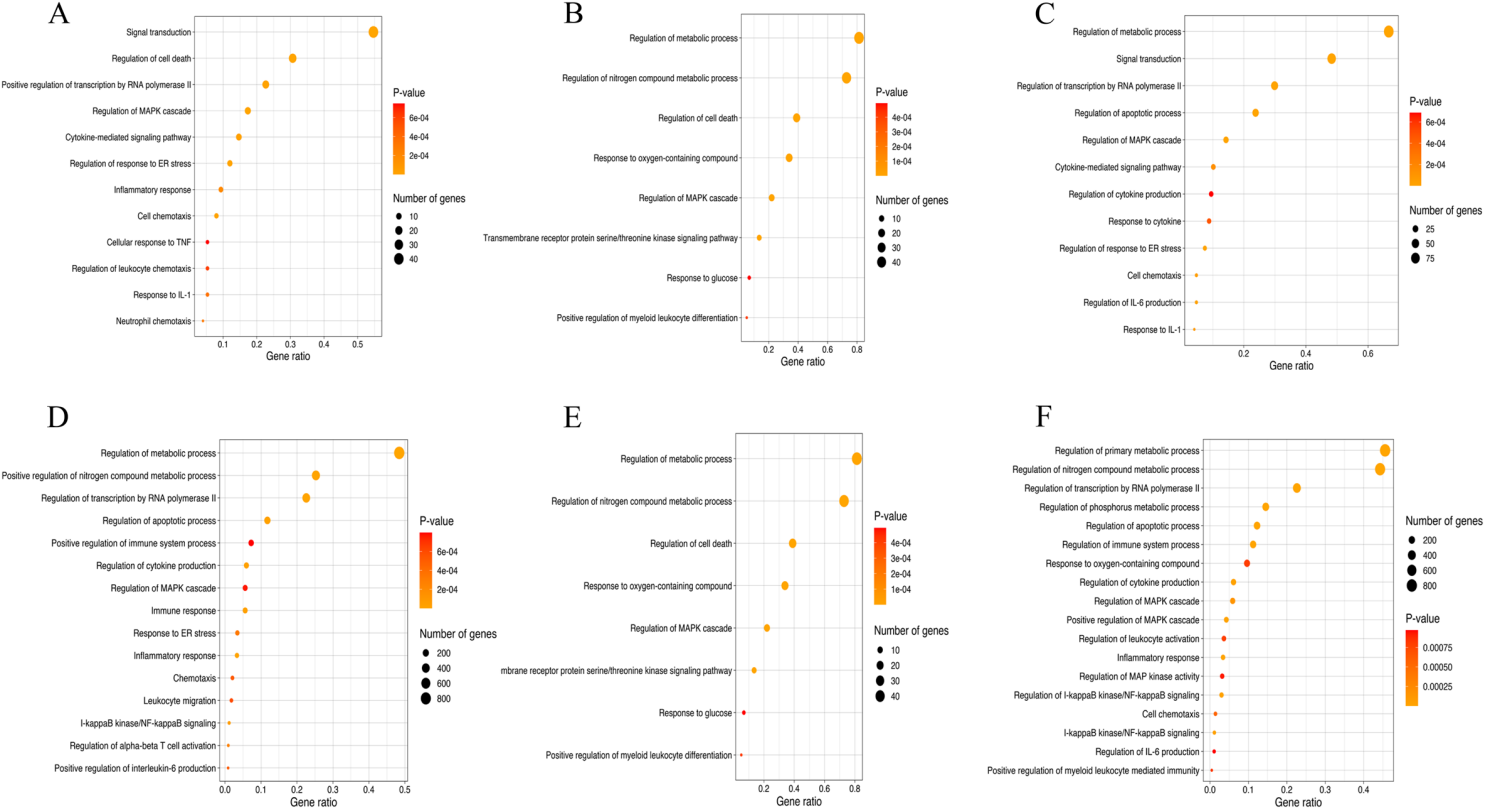
Selected Gene Ontology (GO) terms enriched in the present work among upregulated genes. (**A**) BCoV-infected cells at 24 hpi; (**B**) *C. parvum*-infected cells at 24 hpi; (**C**) Co-exposed cells at 24 hpi; (**D**) BCoV-infected cells at 72 hpi; (**E**) *C. parvum*-infected cells at 72 hpi; (**F**) Co-exposed cells at 72 hpi.

Among the GO terms enriched in our subset of DEGs, it is noteworthy that most of those enriched in BCoV-infected cells at 24 hpi are involved in immune processes, such as cytokine-mediated signalling pathways, inflammatory response, cell chemotaxis, and response to IL-1 (Fig. 4a, Table 3, Supplementary table 4). In contrast, and as reflected in the number of DEGs, the effect of *C. parvum* infection on the host cell immune responses was more limited; the most enriched GO terms *C. parvum*-infected cells at 24 hpi belong to metabolic categories, such as response to purine-containing compounds or hepoxilin biosynthesis (Fig. 4b, Table 3, supplementary table 5). The pattern seen in co-exposed cells is again more like that seen in BCoV-infected cells, with several GO terms related to immune processes. It should be noted that the GO term “regulation of IL-6 production” appears in co-exposed cells and is absent from cells infected only with BCoV at 24 hpi, which could indicate additional immune regulation (Fig. 4c, Table 3). IL-6 has been described as presenting a proinflammatory effect but can also function as a regulatory cytokine^43,44^.

**Table 3:** Gene ontology (GO) terms associated with single-infected and co-exposed cells.

Comparison	BCoV-infected		*C. parvum*-infected		Co-exposed (BCoV + *C. parvum)*	
Post infection time (hpi)	24 hpi	72 hpi	24hpi	72 hpi	24 hpi	72 hpi
Number of GO terms among upregulated genes (Biological process)	243	273	45	96	269	332
Examples	Regulation of signal transduction, cellular response to stress, positive regulation of cell death	Regulation of cellular metabolic process, regulation of nitrogen compound metabolic process, regulation of NF-κ B signalling	Oxidative phosphorylation, respiratory electron transport chain	Regulation of nitrogen compound metabolic process, regulation of MAPK cascade	Regulation of metabolic process, response to ER stress	Regulation of cellular metabolic process, NF-κB signalling
Number of GO terms downregulated genes (Biological process)	–	73	–	9	–	91
Examples	–	SRP-dependent co-translational protein targeting to membrane, protein targeting to ER, etc	–	Regulation of nucleobase-containing compound metabolic process, nucleosome assembly, etc	–	Lipid metabolic process, Oxidative phosphorylation, etc

At 72 hpi, the number of GO terms relating to immune responses was enriched, and the number of DEGs associated with them was higher than at 24 hpi for all set ups (Table 3). For BCoV-infected cells at 72 hpi, there was an enrichment of GO terms related to apoptotic processes, but also many GO terms associated with immune responses. This could indicate a later effect on the host cells after the initial proinflammatory response (Fig. 4d, Supplementary table 5). These GO terms reflect a positive regulation of several signalling cascades, including IL-6 production, inflammatory responses, or the signalling pathway of NF-κB. The fact that the inflammatory response occurs at both time points, but with an increased number of genes involved at 72 hpi, indicates a broadening and strengthening of the inflammatory response. For *C. parvum-*infected cells at 72 hpi, the enriched GO terms remained associated with metabolic processes, but other GO terms, such as cell death and responses to oxygen-containing compounds, also occur (Fig. 4e). Production of reactive oxygen species (ROS) is one of the key mechanisms that host cells have evolved to counteract intracellular parasites^45^, but *C. parvum* is extremely resistant to oxidants^46^. However, GO terms associated with immune processes were also enriched in the *C. parvum*-infected cells, such as “positive regulation of myeloid leukocyte differentiation”. Interestingly, when the host response from *C. parvum*-infected cells was compared with BCoV-infected cells, fewer immune genes were found as regulated; this could be due to *C. parvum* residing in the parasitophorous vacuole, thus evading the host response^22^. However, as mentioned previously, this could also result from fewer host cells being infected by the parasite than by the virus. At 72 hpi, the pattern is similar in co-exposed and BCoV-infected cells, with more GOs enriched that are associated with immune system processes (Fig. 4), such as “regulation of cytokine production”; “regulation of I-kappaB kinase/NF-kappaB signalling” and “interleukin-1 mediated signalling pathway” (Fig. 4f). Among the down-regulated DEGs, for both co-exposed and virus-infected cells at 72 hpi, most of the GO terms belong to metabolic processes, processes involved in lipid metabolism, protein-DNA complex organization, or carboxylic acid metabolism.

#### Infected HCT-8 cells show a marked proinflammatory profile after BCoV or co-infection with BCoV and *C. parvum*, whereas *C. parvum* has a more limited effect

From the enrichment, five KEGG pathways (MAPK, TNF, NF-κB, IL-17 and apoptosis) were selected for exploration in further detail, based on the number of DEGs and the biological significance of the pathways (Table 4, Supplementary tables 4–9, Supplementary Fig. 3).

**Table 4:** KEGG pathways associated with single-infected and co-exposed cells.

Comparison	BCoV-infected	*C. parvum*-infected	Co-exposed	Co-exposed vs BCoV-infected	Co-exposed vs *C. parvum-*infected
Post infection time (hpi)	24 hpi				
Total N pathways enriched	34 (34 for upregulated genes)	9 (11 for upregulated genes)	19 (22 for upregulated genes)	0 for up; 5 for downregulated genes	4 for up; 9 for down
Pathways selected	MAPK, IL-17, TNF, NF-κB		MAPK, IL-17, TNF, NF-κB	NA	IL-17, TNF, MAPK
					Oxidative Phosphorylation
Pathways selected	MAPK, IL-17, TNF, NF-κB	NA	MAPK, IL-17, TNF, NF-κB	NA	IL-17, TNF, MAPK
					Oxidative Phosphorylation
Post infection time (hpi)	72 hpi				
Total N pathways enriched	28 (first 2000 genes upregulated); 15 (first 2000 downregulated genes)	17 (35 for upregulated genes)	42 (first 2000 upregulated genes); 13 for the first 2000 downregulated genes	NA	25 for upregulated genes, 30 for downregulated genes
Pathways selected	MAPK, IL-17, TNF, NF-κB	MAPK, IL-17	MAPK, IL-17	NA	TNF, IL-17, NF-κB

The Mitogen Associated Protein Kinases (MAPK) pathway is involved in many key cellular processes, with variable outcomes such as cell proliferation, differentiation, development, inflammatory responses, and apoptosis. Generally, signalling from this pathway starts when a signalling molecule binds to a receptor in the cell surface and the signalling cascade affects transcription in the nucleus^47^. In the present study, various components of the MAPK signalling pathway were modulated in all experimental set ups. In co-exposed cells, the gene encoding for *MAP3K8* was upregulated at 24 hpi (FC = 1.67). *MAP3K8* has been shown to be required for lipopolysaccharide-induced, toll-like receptor-4 (TLR4)-mediated activation of the MAPK/ extracellular signal-regulated kinase (ERK) pathway in macrophages and is thus critical for production of the proinflammatory cytokine TNF-alpha (TNF-α) during immune responses^48^. It is also involved in the regulation of T-helper cell differentiation and interferon gamma (IFNG) expression in T-cells. In the current study, more genes belonging to MAPK pathway were modulated at 72 hpi (n = 49 and 55) for BCoV-infected and co-exposed cells, respectively. (Table 4). Among these, *MAPK3K8* is upregulated for both (FC = 4.3 and 4.2, respectively), in contrast to previous studies carried out with otherβ-coronaviruses^49^. The relevance and other possible differences in the modulation of this pathway between BCoV and other coronaviruses remain to be explored. As a pleoiotropic pathway, several of the potential outcomes from upregulation of the MAPK pathway is proliferation/differentiation/inflammation and apoptosis through the transcription factor *FOS*, which was upregulated in both BCoV and *C. parvum* single infections. Among downregulated genes, several genes involved in the MAPK pathway were found, such as *MAPK9* (FC = -1.78), *MAPK3* (FC = -2.05), *DAB2IP* (FC = 6.28) (with functions in several pathways implicated on metabolism, proliferation, cell survival, growth, and angiogenesis). In the co-infection setup, *TRAF2* and *CX3C* were upregulated, whereas *RIPK3* was downregulated.

In our study, MAPK modulations were not seen in *C. parvum*-infected cells, and the pathway was not found to be significantly enriched at 24 hpi (Supplementary table 3). Nevertheless, at 72 hpi, the pathway was enriched, and several genes involved in this pathway, such as *GADD45B* (FC = 1.94)*, DUSP1* (FC = 4.91)*, DUSP6* (FC = 1.5)*, DUSP5* (FC = 1.65)*, FOS* (FC = 5.18)*, JUN* (FC = 3.85) and *IGF2* (FC = 1.55), were upregulated (Supplementary table 3. Dual specificity phosphatases (DUSPS) have been described as anti-cancer molecules, being inhibitors of cell proliferation and MAPK activation^50^. A study on murine intestinal epithelial cells infected with *C. parvum* showed that most of the genes encoding the key components of the MAPK signalling pathway were not modulated. However, a significant downregulation of *p38*/*Mapk*, MAP kinase-activated protein kinase 2 (*Mk2*), and *Mk3* genes was found. This suppression of MAPK signalling activity in *C. parvum*-infected intestinal epithelial cells was postulated as a strategy to evade host immune response^51^.

Interlinked with the MAPK signalling cascade, it is worth noting that the pathway for tumour necrosis factor alpha (TNF-α) was enriched in all our experimental setups, except for *C. parvum* infected cells at 24 hpi (Supplementary tables 6–7, supplementary Figs. 5–6). TNF-α is a proinflammatory cytokine produced upon activation of the immune system and involved in various biological processes, including regulation of cell proliferation, differentiation, apoptosis, and immune responses^52^. Several genes involved in TNF signalling were modulated in our experimental setups (supplementary tables 4–9, supplementary Figs. 5–6). At 24 hpi, several genes were upregulated in BCoV-infected cells at 24 hpi, including genes coding for cytokines, such as IL-1A (FC = 12.14), IL-8 (FC = 13.95), *FOS* (F = 4), and *CREB5* (FC = 2.8). It is noteworthy that also genes coding for several chemokines, such as *CXCL2* (FC = 8), also termed *GROβ* (Growth-regulated protein beta) which is involved in immunoregulatory and inflammatory processes acting as a chemotactic for neutrophils; and CXCL3 (FC = 2.56), were upregulated (Supplementary Fig. 5).

One of the signalling cascades after TNF-α involves the nuclear factor kappa-light-chain-enhancer of activated B cells (NF-κB) activation. In turn, NF-κB activation induces transcription and expression of genes encoding proinflammatory cytokines, such as IL-6, but also anti-apoptotic factors such as BIRC2, BIRC3, and BCL-2 homologue BCL2L1, enabling the cell to remain inert to apoptotic stimuli^53^.

The importance of NF-κB in several viral and parasitic diseases has been extensively reported^54,55^. It has been shown to be responsible for the rapid induction of type I IFNs (such as IFN-β) and other proinflammatory cytokines (e.g., IL-6 and IL-8) during infections with RNA virus^56,57^. It has been suggested that several TLRs, such as TLR2, TLR3, TLR4, TLR7/8, and TLR9, contribute to antiviral responses against infections caused by coronaviruses, both located in the cell membrane and intracellular^58^. Amongst them, TLR3 and TLR7 are capable to sense ssRNA in the endosomes, and dsRNA can be also recognised by cytosolic RIG-like receptors (RLRs) in human epithelial cells^59^. This activates the interferon regulatory factor (IRF) IRF3, IRF7, and NF-κB, which are key regulators of proinflammatory response and innate immunity^60^. In BCoV-infected cells, several genes involved in the NF-κB pathway were upregulated at 24 hpi, such as *PTGS2* (FC = 1.72); *CXCL2* (FC = 8); *IL8* (FC = 13.95). *PTGS2* represents an inducible prostaglandin-endoperoxide synthase involved in inflammatory processes by regulating the synthesis of prostaglandins^60^.

In *C. parvum*-infected cells, activation of NF-κB, induces the expression of pro- and anti-apoptotic factors (Liu et al., 2009) and proinflammatory cytokines (e.g., TNF-α, IL-8)^61–64^. TNF-α and TGF-β play roles in providing the host protective immunity and tissue repair effects against infection^65^. In conjunction with ERK1/2 and p38 MAPK pathways, host cells attempt to destroy the parasite by inducing formation of the neutrophil extracellular traps (NETosis)^66^. In addition, multiple genes, such as heat-shock genes, chemokines (such as *CXCL8, CCL5, CXCL10, SCYB5*), host actin and tubulin, are known to be upregulated in *C. parvum* infection in human epithelial cells, presumably in an attempt to eliminate the pathogen^67,68^. However, none of these genes were found to be modulated in *C. parvum*-infected cells in our study. At 72 hpi, transcription factors *FOS* (FC = 5.18) and *JUN* (FC = 3.85) were upregulated.

In the co-infection setup, *TNFAIP3* was upregulated at 24 hpi (FC = 1.64). This transcription factor is involved in immune and inflammatory responses signalled by cytokines, such as TNF-α and IL-1 beta, or pathogens via TLRs through termination of NF-κB activity, ensuring the transient nature of inflammatory signalling pathways^69^.

At 72 hpi, the modulation was more evident, with more genes belonging to inflammatory pathways being differentially expressed (Supplementary tables 4–9, Figs. 7–8). In BCoV-infected cells, several genes were upregulated that are responsible for leukocyte recruitment and activation, such as chemokines (*CXCL2*, *CXCL5*, FC = 139 and 6.54, respectively), genes responsible for the activation of immune cells (*CSF1,* FC = 4.06); surface receptors (*JAG1,* FC = 2.46); *MAPK6* (FC = 3.10); *BIRC2* (FC = 3.17); *RIPK1* (FC = 2.68), involved in the necrosome); molecules responsible for cell adhesion: *ICAM1* (FC = 3.98); Vascular factors: *EDN1* (FC = 67,97); *TRAF3* (FC = 2.59); *CHUK* (FC = 2.19); *IKBKG* (FC = 5); *NF*-*κB1* (FC = 4.5)/ *NF*-*κBIA* (FC = 11.77)*, NFKBIB* (FC = 9.42)*, NFKBIE* (FC = 8.87)*, NFKBIZ* (FC = 4.62) (NF-κB inhibitors); *ATF4* (FC = 1.8); *CEBPB* (FC = 3.55); *IRF1* (FC = 3.57).

Also, in co-exposed cells at 72 hpi, there was an upregulation of genes coding for sensors of DNA damage such as *IKBKG* (FC = 3.98), *UBE21* (sumoylation), *RIPK1* (FC = 2.8); inflammation (*DDX26B,* FC = 3.72) and canonical and non-canonical pathways for hypoxia. Several of these genes converge in the degradation of the inhibitor NF-κBIA, activating the transcription of target genes. Downregulated genes in BcoV-infected cells at 72 hpi included *EDA* (FC = -2.5)/*EDAR* (FC = -6.74), which mediate the activation of NF-Κb and JNK; surface marker *CD14* (FC = -4.57), which acts via MyD88, *TRAPPCC1* and *TRAF6*, leading to NF-κB activation, cytokine secretion, and the inflammatory response^70^. In addition, *TRAPPCC1* was also downregulated (FC = -2.05). This is an adapter involved in TLR2 and TLR4 signalling pathways in the innate immune response, resulting in cytokine secretion and inflammatory responses, positively regulating the production of TNF-α and IL-6.

Interleukin 17 is a family of proinflammatory cytokines that act as potent mediators in delayed-type reactions by increasing chemokine production in various tissues in response to extracellular pathogens, acting synergistically with TNF and IL-1^71^. Several genes involved in this pathway were modulated in the present study (Supplementary tables 4–9; Supplementary Figs. 9–10). Its relevance has been recently explored in other viral diseases, including COVID-19^72^. Furthermore, a few studies have shown that it is important in cryptosporidiosis, with a rapid induction in the intestine of mice and bovines^73,74^. At 24 hpi, the genes involved in this pathway and that were upregulated in BcoV-infected cells included *FOS (FC* = *2)*, *CXCL2 (FC* = *8)*, CXCL3 (FC = 2.56), IL-8 (FC = 13.95) and *PTGS2 (FC* = *1.72)*. These chemokines and cytokines may lead to autoimmune pathology, neutrophil recruitment, and immunity against extracellular pathogens. The pathway was not modulated in *C. parvum* infected cells at 24 hpi (Suplementary table 6, Supplementary Fig. 7). In the co-exposed cells, aside from genes such as CXCL2 (FC = 9.24), CXCL3 (FC = 3.23) and CXCL8 (FC = 15), the gene *TNFAIP3* was upregulated (FC = 1.64), which is induced by TNF-α. This mechanism could inhibit NF-κB activation and apoptosis mediated by TNF-α and limit inflammation. Two other genes, *FOSB* (FC = 3.44) and *MAPK6 (F* = *1.54)*, were also upregulated in co-exposed cells at 24 hpi, but were not differentially expressed in either of the single infections for the same time point.

More extensive modulation was evident in BcoV-infected cells at 72 hpi, where several genes were upregulated, including several chemokines such as CXCL1 (FC = 44.97), CXCL2 (FC = 139), CXCL3 (FC = 43.64), CXCL5 (FC = 6.55), and matrix metalloproteases, such as *MMP1* (FC = 6.31)and *MMP13 (FC* = *40.85)*, which are important for the remodelling of extracellular matrix. Matrix metalloproteases, induced after the expression of proinflammatory cytokines, contribute to pathogenesis by disrupting the barrier function of cells, and have been shown to be relevant in other viral diseases^75^. The damage to the barrier function of enterocytes and intestinal cells could lead to diarrhoea, the clinical sign most associated with both BcoV and *C. parvum* infection in vivo.

Apoptosis is characterized by a series of dramatic perturbations to the cellular architecture that contribute not only to cell death, but also prepare cells for their removal by phagocytes. This prevents the occurrence of unwanted immune responses and is commonly associated with both viral infections and parasitic infections^76,77^. During the execution phase of apoptosis, several mechanisms are orchestrated by the caspase family of cysteine proteases. Caspases (CASP) target proteins for restricted proteolysis in a controlled manner, minimizing damage and the release of immunostimulatory molecules^78^. Interestingly, in our experimental setup, BCoV-infected and co-exposed cells at 72 hpi show a balance between the upregulation of pro-apoptotic genes, such as *BAK1*(FC = 2.63 and 2.62, respectively), *TNFSRF10B* (fc = 2.92 and 2.83, respectively), *PMAIP1* (fc = 3.67 and 3, respectively), and pro-survival genes such as *BIRC-2,* FC = 3.18 and 2.86, respectively); in addition, there was downregulation of pro-apoptotic genes such as *BBC3* (FC = -6.91 and -9.75, respectively). Among the key apoptotic genes, *CASP7* was upregulated in BcoV and co-exposed cells at 72 hpi (FC = 4.12 and 3,78, respectively) (Supplementary tables 4–9 supplementary Figs. 11–12). This caspase has been shown to have a role in inflammation, being activated by caspases-8 and -9 and presenting the same function as *CASP3*^79^. Genes coding for other caspases, such as *CASP8AP2* (involved in the tnf-α induced activation of NF-Κb) (FC = -1.68 and -1.87), *CASP5* (initiation of pyroptosis, regulation of antiviral innate immune activation) (FC = -10.26 and -7.32, respectively), *CASP6* (FC = -2.81 and -2.7 respectively) were downregulated in both BCoV-infected and coinfected cells. *CASP2* (involved in the initiator phase) (FC = -1.90), and *CASP4* (involved in inflammation) (FC = -1.74) were downregulated after 72 hpi only in co-exposed cells. Also, B-cell lymphoma (BCL) class genes were mostly upregulated (such as BCL6 (FC = 20.16 and 17.59) and BCL10 (FC = 4.17 and 3.65), while cellular tumour antigen p53 was downregulated in both BcoV-infected and co-exposed cells at 72 hpi (FC = -1.57 and -1.60). Other BCL genes, such as *BCL7c* (FC = -2.26 and -2.13), *BCL11A* (FC = -4.75 and -12.4), , and *BCL2L14* (FC = -7.3 and -5.7), were also downregulated in both set ups. Cellular apoptosis has been described to occur as a host response to evade parasite invasion in the early phase of *C. parvum* infection^80,81^. A microarray analysis of infected host intestinal mucosa has revealed overexpression of TNF-superfamily receptor osteoprotegrin (OPG), which inhibits the TNF-α-related-apoptosis-inducing ligand (TRAIL)-mediated apoptosis^82^. This helps the parasite to escape the host defences and complete its life cycle. However, in our experiments, only the genes coding for *GADD45B* (FC = 1.94), *FOS* (FC = 5.18), and *JUN* (FC = 3.85) were modulated at 72 hpi in *C. parvum*-infected cells.

#### The proinflammatory modulation found in vitro could indicate the basis for the molecular pathogenesis and intestinal damage in vivo

As hinted before, several upregulated genes following BcoV and co-infection are involved in proinflammatory responses. Some of these genes are implicated in IFN-I induction and signalling at 24 and 72 hpi in BcoV-infected cells. Despite the importance of Interferon Stimulate Genes (ISGs), only the gene coding for ISG20 was differentially expressed at 72 hpi, being upregulated in both BCoV-infected and co-exposed cells (FC = 3.55 and 3.73, respectively), together with the gene coding for ISG20L2 (FC = 2.55 and 2.31, respectively). ISG20 is an interferon-induced antiviral exoribonuclease that acts on single-stranded RNA and exhibits antiviral activity against RNA viruses, including hepatitis C virus (HCV), hepatitis A virus, and yellow fever virus, in an exonuclease-dependent manner^83,84^andthe pattern of expression of ISGs was almost identical in BcoV-infected and co-exposed cells, with only one difference: *OAS3* (FC = 1.3), which was found in co-exposed cells, but not in BcoV-infected cells at 72 hpi. was. IFITM10 was upregulated in BCoV-infected cellsIn contrast, genes coding for other interferon-responsive elements, such as, *IFITM2* (FC = -4.7 and -2.8), *IFITM3* (FC = -3–38 and -3.87), and *IFI27* (FC = -3.62 and -3.38), were downregulated in both set ups. The gene coding for *IFITM1* (FC = -1.80) was downregulated in BCoV-infected cells, but not in co-exposed cells*.* The gene coding for *IL-1A*, considered as one of the endogenous pyrogens and a potent proinflammatory protein, was also upregulated at both 24 (FC = 12.14 and 15.78) and 72 hpi (FC = 46.7 and 41.45) for both BCoV-infected and co-exposed cells, together with tumour necrosis factor receptors (TNFRs), *TNFRSF12A* (FC = 1.62 and 1.88 at 24 hpi; 10.1 and 8.5 at 72 hpi, and chemokines such as *CXCL2* (8 and 9.24 t 24 hpi, 139 and 131 at 72 HPI, *CXCL8 (IL8*) (13.95 15 at 24 hpi, 323 and 268), and *CXCL3*.(2.59 3.21 at 24 hpi 44.87 30.82, respectively* )*.

At 72 hpi, we found a modulation of both anti-inflammatory and proinflammatory genes, which might represent an attempt of the host cells to counteract BcoV infection. TLRs showed a mixed expression profile, since *TLR3* (FC = -7.2 and -5.3) and *TLR5* (FC = -3.7 and -3.4) were downregulated, while *TLR6* was upregulated (FC = 6 and 5.47) in both BCoV-infected and co-exposed cells. *TLR3* is a nucleotide-sensing TLR that is activated by double-stranded RNA, a sign of viral infection, associating endosomal recognition of viruses to IFN-I responses and leading to NF-κβ activation, cytokine secretion, and the inflammatory response^85,86^. However, this receptor probably does not have a function during the initial steps of infection with BcoV, but later during the replication. Genes coding for TNFR receptors, such as TNFRSF10A (FC = 1.62 and 1.6), TNFRS10B (FC = 2.92 and 2.83), TNFRS10D (FC = 3.23 and 2.78) and TNFRS18 (FC = 2.92 and 2.7) were only upregulated at 72 hpi in both BCoV and co-exposed cells. In addition, *DDX58* (FC = 3.25 and 3.07*), OASL* (FC = 14.9 and 14.02, respectively), and *ISG20* were upregulated in both BCOV and co-exposed cells. *DDX58* (also known as RIG-1) is an immune receptor that senses cytoplasmic viral nucleic acids and activates a downstream signalling cascade leading to the production of type I IFNs and proinflammatory cytokines^87^. OASL (2’-5’-oligoadenylate synthetase-like protein) displays antiviral activity against encephalomyocarditis virus and HCV via an alternative antiviral pathway independent of Rnase L^88^. Also, the genes coding for *MT2A* (metallothioneins 2) (fc = -3.36 and -4.22) and *GBP3* (FC = -2.31 and -2.13) (guanylate-binding protein 3) were downreguated in co-exposed cells at 72 hpi. Furthermore, modulation of host cytoskeleton activities was found, with upregulation of lectins (*CLEC4,* FC = 9.23 and 9.89) and *IKBKG* (FC = 5 and 3.98) in BcoV-and co-exposed cells at 72 hpi. In co-exposed cells at 24 hpi, heat shock protein 90 (hsp 90) was upregulated (FC = 1.60), while keratin type I cytoskeletal 20 (KRT20) was downregulated (FC = -1.54). Similarly, in both BCoV infected and co-exposed cells at 72 hpi, genes coding for mucins (such as *MUC13* (FC = 2.09 and 1.59), and *MUC4* (FC = 9 and 9.91)) were upregulated. The gene coding for MUC3A was downregulated in BCoV cells (FC = -1.64), whereas MUC20 was upregulated (FC = 2.72) in coinfected cells. Pathogens damaging the gastrointestinal tract cause damage to the mucus barrier, which can worsen mucus quality and reduce mucus production, potentially leading to chronic inflammation and disease^89^.

Taking all our findings together, the proinflammatory modulation, in concert with the alterations in the host cell cytoskeleton and the protective mucus barrier, could suggest the molecular basis in vivo for damage to the epithelial barrier, leading to diarrhoea. In vivo*,* both BCoV and *C. parvum* replicate in enterocytes, causing morphological changes in the intestinal cytoskeleton, such as loss of microvilli and shortening of columnar epithelium, thereby causing severe villous atrophy. This leads to reduced digestion of feed and decreased absorptive capacity of the intestine, causing an osmotic imbalance that could be exacerbated by changes in the permeability of the epithelial cells^90,91^. Additionally, it has been recently shown that concentrations of IL-8 in serum from BCoV infected calves increased from 0 to 24 hpi, indicating intestinal injury and diarrhoea^92^. However, our results provide no indication of a greater severity when both pathogens are present, as the host expression profiles between single BCoV-infection and co-infection are very similar. As mentioned before, this could be due to the fact that the infection rate achieved by the virus is higher than for *Cryptosporidium parvum*. Moreover, the BCoV strain employed had been adapted to the HCT-8 previously, so this adaptation could influence the viral properties in vitro, and, possibly, the host–pathogen interactions. Studies on bovine coronavirus are lacking, but it has been previously shown that in vitro adaptation to new host environments causes mutations and selection of different genotypes^93^

In the present study, we have chosen a human cell line, so the possible relevance and influence on pathogenesis in the target species (bovine) need to be explored by using more complex in vitro models or by in vivo approaches. In this scenario, bovine intestinal organoids (enteroids) have been recently described to be permissive for bovine coronavirus^28^ and represent a more complex system, comprising different cell populations (such as goblet cells, Paneth cells and enteroendocrine cells), which could better relate to the in vivo situation.

#### Genes exclusively expressed in co-exposed cells as potential biomarkers for BCoV and *C. parvum* co-infection

At 24 hpi, 78 DEGs were identified in co-exposed cells, with the highest FC corresponding to *NR4A3* (FC = 4.12), a transcriptional activator involved in regulating proliferation, survival differentiation, and inflammatory processes^94^. At 72 hpi, 803 DEGs were uniquely expressed in co-exposed cells (Fig. 3). The highest FC corresponds to *DNAH17* (FC = 55.20), although the function of this dynein gene in our context is not obvious. GO term analysis mostly showed an effect on cellular metabolic pathways and response to DNA damage both at 24 and 72 hpi (Supplementary table S10). Strikingly, we found no significantly enriched KEGG pathways and few immune genes among these DEGs. Despite this, the high fold change shown by some of the genes could imply that they represent interesting targets as biomarkers for co-infections, and their putative role in the pathogenesis of these intestinal infections should be addressed in in vivo models.

## Conclusions

Our work demonstrates extensive modulation of the host-cell transcriptome by BCoV, influencing immune processes and metabolic pathways, and a more limited effect from *C. parvum* under our experimental settings. The modulation during co-infection seems to be dominated by BCoV, although it should be recognized that there is a possible bias due to the difference in infection success shown by the pathogens. Nevertheless, our findings provide insights into the molecular pathogenesis of these intestinal infections and suggest possible biomarkers associated with co-infection. The function and relevance of these putative genes could be explored further using novel in vitro models (employing bovine intestinal epithelial cells or as bovine intestinal organoids) or in vivo*,* to compare with the results obtained here with this human cell model.

### Supplementary Information

Supplementary Information 1.

Supplementary Information 2.

Supplementary Information 3.

Supplementary Information 4.

Supplementary Information 5.

Supplementary Information 6.

Supplementary Information 7.

Supplementary Information 8.

Supplementary Information 9.

Supplementary Information 10.

Supplementary Information 11.

Supplementary Information 12.

Supplementary Information 13.

Supplementary Information 14.

Supplementary Information 15.

Supplementary Information 16.

Supplementary Information 17.

Supplementary Information 18.

Supplementary Information 19.

Supplementary Information 20.

Supplementary Information 21.

Supplementary Information 22.

## Data Availability

The RNA sequencing data used and analysed in the current study are available from GEO, under the accession number GSE223548.
