# Supplementary material for: Gene expression profile of HCT-8 cells following single or co-infections with Cryptosporidium parvum and bovine coronavirus
Source: Sci Rep. 2023 Dec 13;13:22106. doi: 10.1038/s41598-023-49488-1 (PMC10719361; doi:10.1038/s41598-023-49488-1)
Supplement: Supplementary file 1 — Supplementary Information 1. [file 41598_2023_49488_MOESM1_ESM.pptx]

## Slide 1
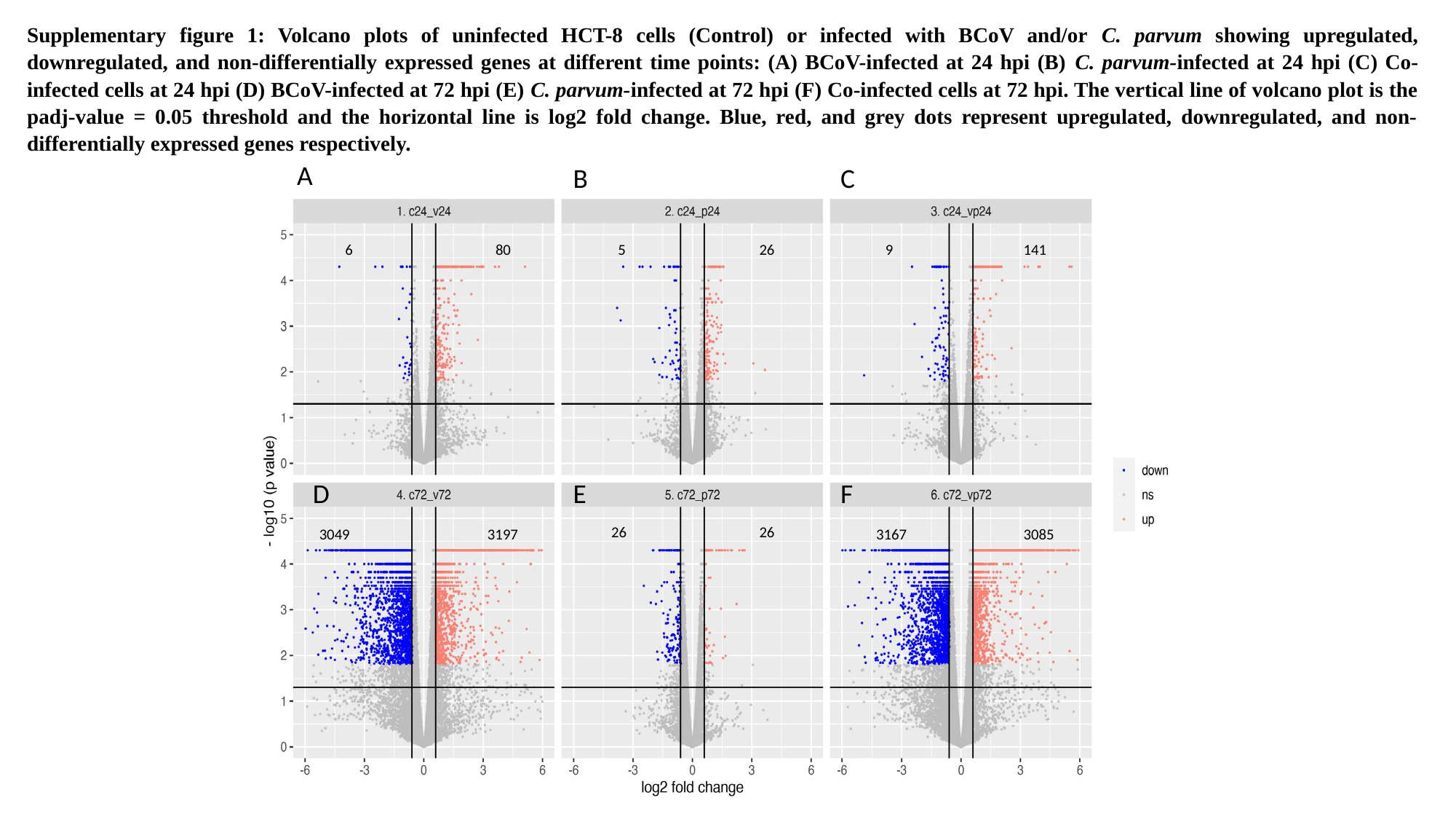

Supplementary figure 1: Volcano plots of uninfected HCT-8 cells (Control) or infected with BCoV and/or C. parvum showing upregulated, downregulated, and non-differentially expressed genes at different time points: (A) BCoV-infected at 24 hpi (B) C. parvum-infected at 24 hpi (C) Co-infected cells at 24 hpi (D) BCoV-infected at 72 hpi (E) C. parvum-infected at 72 hpi (F) Co-infected cells at 72 hpi. The vertical line of volcano plot is the padj-value = 0.05 threshold and the horizontal line is log2 fold change. Blue, red, and grey dots represent upregulated, downregulated, and non-differentially expressed genes respectively.
A
B
C
26
141
6
80
5
9
26
26
3197
3049
3085
3167
D
E
F
