# Supplementary material for: Gene expression profile of HCT-8 cells following single or co-infections with Cryptosporidium parvum and bovine coronavirus
Source: Sci Rep. 2023 Dec 13;13:22106. doi: 10.1038/s41598-023-49488-1 (PMC10719361; doi:10.1038/s41598-023-49488-1)
Supplement: Supplementary file 2 — Supplementary Information 2. [file 41598_2023_49488_MOESM2_ESM.pptx]

## Slide 1
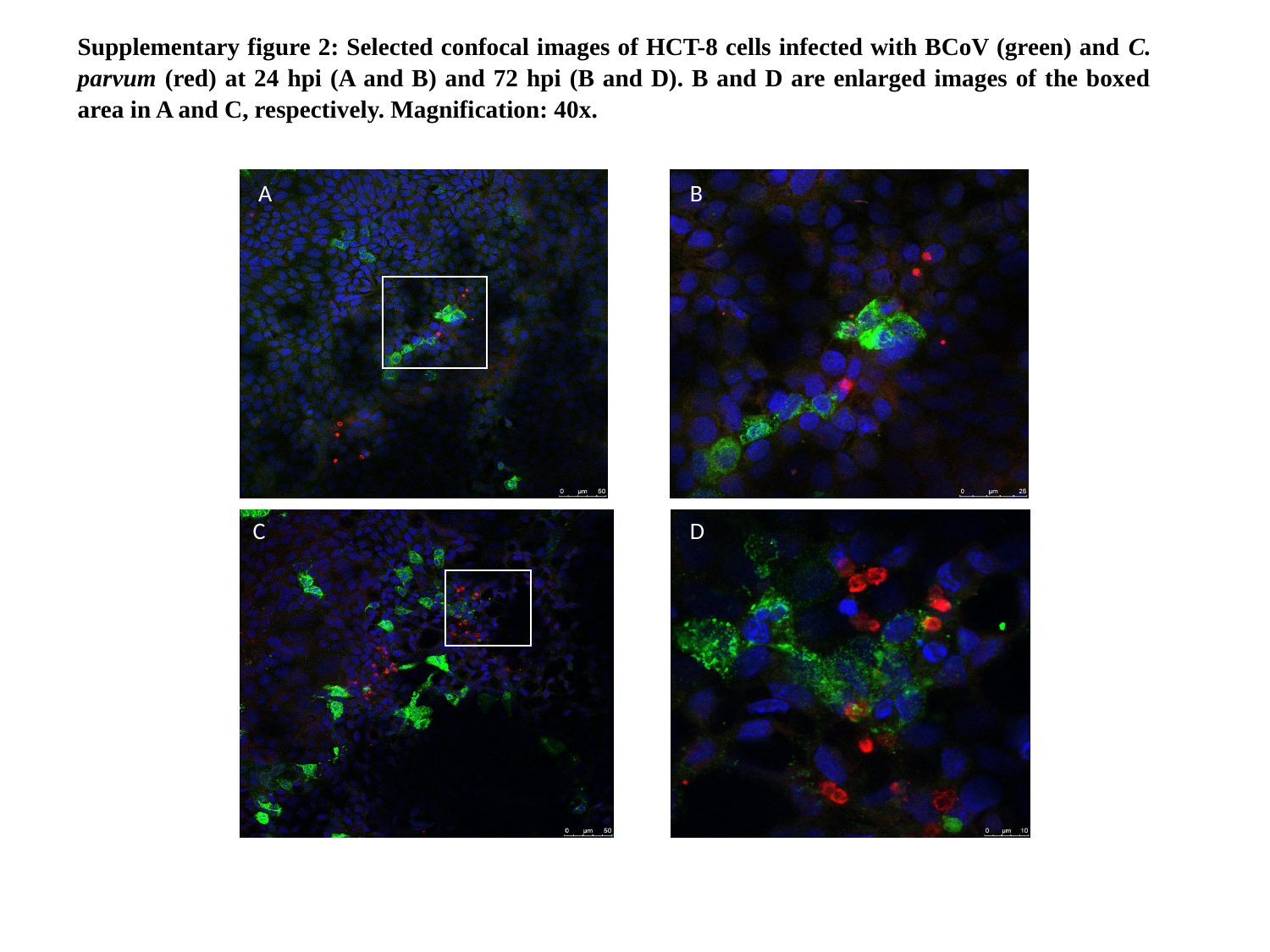

Supplementary figure 2: Selected confocal images of HCT-8 cells infected with BCoV (green) and C. parvum (red) at 24 hpi (A and B) and 72 hpi (B and D). B and D are enlarged images of the boxed area in A and C, respectively. Magnification: 40x.
A
B
C
D
