# Supplementary material for: Gene expression profile of HCT-8 cells following single or co-infections with Cryptosporidium parvum and bovine coronavirus
Source: Sci Rep. 2023 Dec 13;13:22106. doi: 10.1038/s41598-023-49488-1 (PMC10719361; doi:10.1038/s41598-023-49488-1)
Supplement: Supplementary file 3 — Supplementary Information 3. [file 41598_2023_49488_MOESM3_ESM.pptx]

## Slide 1
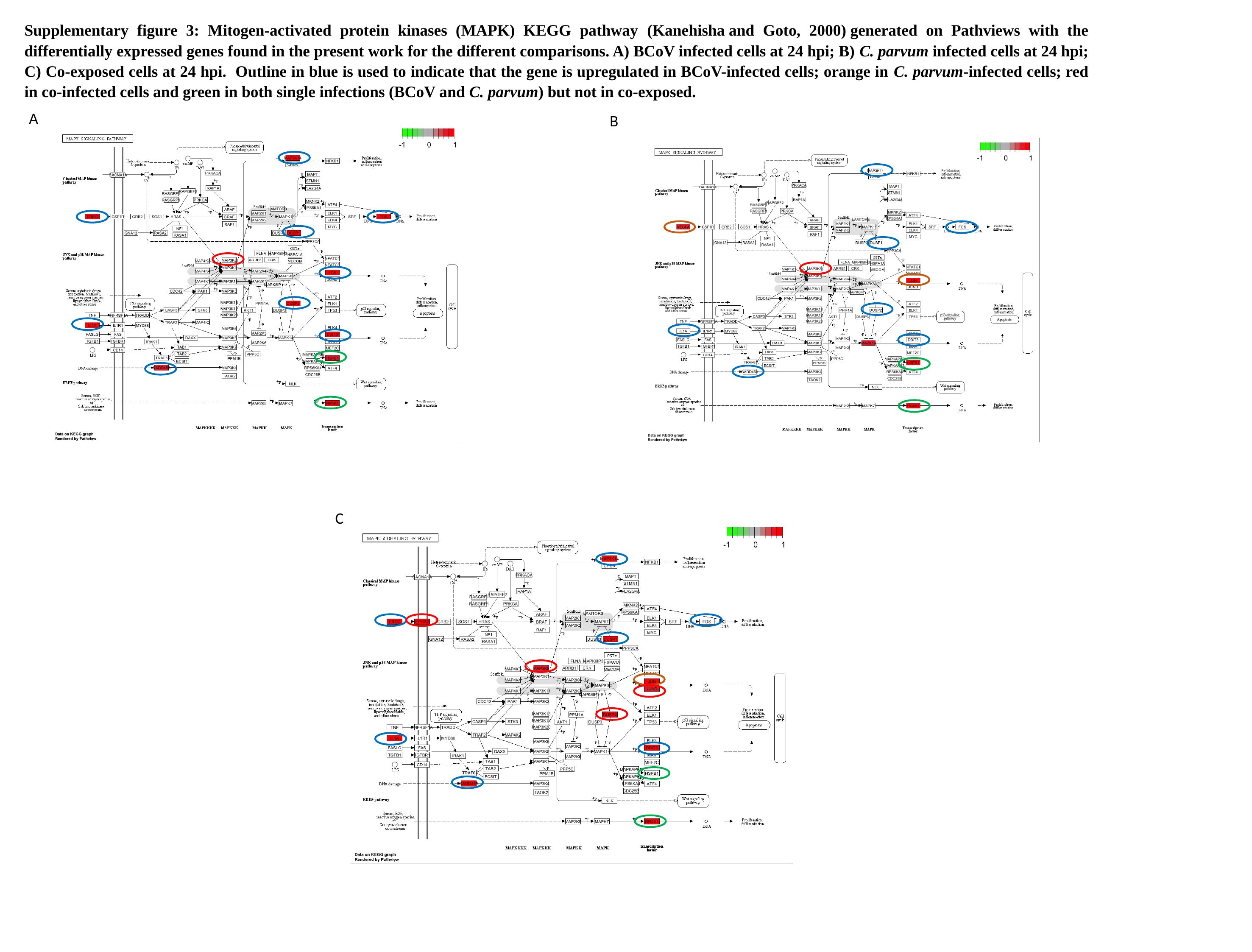

Supplementary figure 3: Mitogen-activated protein kinases (MAPK) KEGG pathway (Kanehisha and Goto, 2000) generated on Pathviews with the differentially expressed genes found in the present work for the different comparisons. A) BCoV infected cells at 24 hpi; B) C. parvum infected cells at 24 hpi; C) Co-exposed cells at 24 hpi. Outline in blue is used to indicate that the gene is upregulated in BCoV-infected cells; orange in C. parvum-infected cells; red in co-infected cells and green in both single infections (BCoV and C. parvum) but not in co-exposed.
A
B
C
