# Supplementary material for: Gene expression profile of HCT-8 cells following single or co-infections with Cryptosporidium parvum and bovine coronavirus
Source: Sci Rep. 2023 Dec 13;13:22106. doi: 10.1038/s41598-023-49488-1 (PMC10719361; doi:10.1038/s41598-023-49488-1)
Supplement: Supplementary file 7 — Supplementary Information 7. [file 41598_2023_49488_MOESM7_ESM.pptx]

## Slide 1
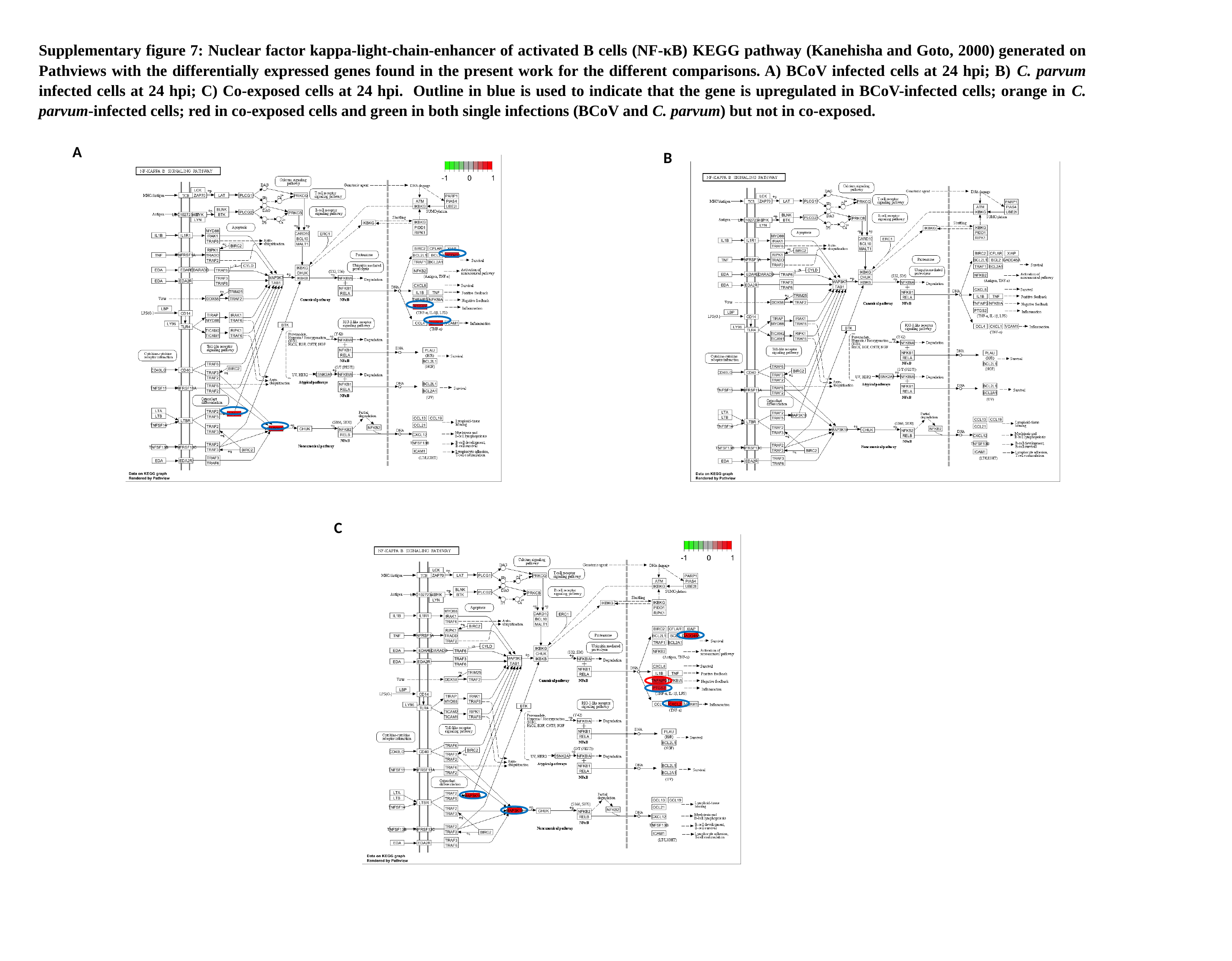

Supplementary figure 7: Nuclear factor kappa-light-chain-enhancer of activated B cells (NF-κB) KEGG pathway (Kanehisha and Goto, 2000) generated on Pathviews with the differentially expressed genes found in the present work for the different comparisons. A) BCoV infected cells at 24 hpi; B) C. parvum infected cells at 24 hpi; C) Co-exposed cells at 24 hpi. Outline in blue is used to indicate that the gene is upregulated in BCoV-infected cells; orange in C. parvum-infected cells; red in co-exposed cells and green in both single infections (BCoV and C. parvum) but not in co-exposed.
A
B
C
